# An Eye-Tracking Study of Pain Perception Toward Faces with Visible Differences

**DOI:** 10.3390/bs16010098

**Published:** 2026-01-12

**Authors:** Pauline Rasset, Loy Séry, Marine Granjon, Kathleen Bogart

**Affiliations:** 1 Univ Rennes, Université Rennes 2, LP3C (Laboratoire de Psychologie: Cognition, Comportement, Communication)-UR1285, F-35000 Rennes, France; 2 Laboratoire de Psychologie de Caen Normandie (LPCN UR 7452), Université de Caen Normandie, F-14000 Caen, France; 3 Laboratoire de Psychologie Epsylon, Université de Montpellier Paul Valéry, F-34000 Montpellier, France; marine.granjon@univ-montp3.fr; 4 School of Psychological Science, Oregon State University, Corvallis, OR 97331, USA; kathleen.bogart@oregonstate.edu

**Keywords:** visible difference, pain evaluation, eye-tracking, gaze behavior, public stigma

## Abstract

This research examines the underlying processes of public stigma toward visible facial differences (VFDs) by focusing on gaze behavior. Past research showed that a VFD influences the visual processing of faces, leading to increased attention to the VFD area at the expense of internal features (i.e., eyes, nose, mouth). Since these features primarily convey affective information, this pre-registered study investigates whether this bias also affects pain perception. In an eye-tracking task, participants (*N* = 44) viewed faces that either did or did not display a VFD located in a peripheral area of the face, and that either did or did not express pain, while their gaze behavior was being recorded. Participants then rated perceived pain intensity for each face. Results showed that VFDs diverted attention toward peripheral features and away from internal, pain-relevant features of the face. Surprisingly, participants rated faces with VFDs as experiencing more pain, regardless of whether pain was actually expressed. This suggests that, despite gazing less at facial expressions, observers inferred pain based on task-irrelevant features, likely stereotypes related to the VFD. These findings provide insights into how people with VFDs are perceived and how their emotions are interpreted.

## 1. Introduction

Visible facial differences (VFDs) refer to a range of visible conditions, whether they be congenital (e.g., cleft lip) or acquired (e.g., scars), that impact appearance (Harcourt et al., 2025). These facial differences diverge from both beauty standards and social expectations of facial features. Due to what could be perceived as diverging from the norm, VFDs are still frequently negatively viewed by others, potentially leading to social difficulties (e.g., see Sarwer et al., 2022). To address these difficulties, traditional research has often focused on individual psychological adjustment (i.e., how these individuals cope with VFDs), overlooking the role of public reactions to VFDs. So as to better integrate the importance of social challenges in the lived experiences of people with visible differences, a recent narrative review called for moving beyond an individual lens towards a socio-structural lens (Harcourt et al., 2025). In line with this recommendation, this study draws on a social stigma framework (Bos et al., 2013; Goffman, 1963) to investigate public reactions to VFDs from the perspective of people who do not have a VFD.

Stigmatization encompasses the range of reactions to a stigma, i.e., a condition that is socially considered to be negative (Bos et al., 2013). Empirical evidence coming from various sources (i.e., self-, family, or public report) consistently shows that people with VFDs experience stigmatization (e.g., intrusive behaviors, avoidance; for a review, see Rasset et al., 2022b). As a stigma characterized by high visibility (Pachankis et al., 2018), experiences of VFDs are often marked by unwanted stares and social attention (Bogart et al., 2022). Unlike the general population, people with VFDs do not benefit from “civil inattention” (Macgregor, 1990).

The development of eye-tracking research has made it possible to directly investigate the oculomotor behavior of observers gazing at portraits of people with VFDs. Evidence consistently shows that, compared with equivalent faces without VFDs, observers direct their gaze toward VFDs more quickly and for longer durations (for a systematic review of eye-tracking studies, see Asaad et al., 2020). In addition, when the difference is located in the peripheral area of the face, observers stare for a shorter duration at the internal features of the face (i.e., eyes, nose, mouth; Rasset et al., 2022a). Some research shows that this modified pattern of gaze behavior towards visibly different faces is involved in stigmatizing reactions, such as evaluations (Madera & Hebl, 2012, 2019) and affective reactions (Rasset et al., 2022a; Stone & Potton, 2019).

The behavioral gaze pattern observed in response to VFDs can be explained by attentional capture, a phenomenon in which objects receive prioritized attentional processing even when they are not task-relevant (see Theeuwes, 2025). Accordingly, VFDs would attract observers’ gaze because they are perceptually distinctive, novel, unexpected, and unique facial features (Boutsen et al., 2018). As a result, this over-attention to the VFD would leave little room for the processing of other individual information, thereby biasing evaluations and behavior. For instance, Madera and Hebl (2012) showed that visually focusing on the VFD of an applicant during a video-recorded interview biased the memorization of relevant job-related information verbally provided by the target, which in turn impeded the employability evaluation of the applicant. As such, this research demonstrated the negative impact of over-attending, and more generally over-gazing, at VFDs on the processing of task-relevant individual information provided in parallel.

Adding to these previous findings, the purpose of the present study was to investigate the impact of VFDs on the visual processing of other task-relevant directly available facial information. Among individual cues, affective information, such as emotional expressions, is particularly relevant because it provides critical information about a person’s intentions, feelings, and social states (Brosch et al., 2013; Hareli & Hess, 2012; Zadra & Clore, 2011). Previous findings demonstrated that a peripherally located VFD captures an observer’s gaze but also diverges it away from the internal features of the face (Rasset et al., 2022a). Since the internal features of the face carry important social information such as the emotional state (Young & Bruce, 2023), we predict that perceivers would also display biased processing of the facial emotions expressed by people with VFDs. Thus, the present study seeks to extend prior research by examining the impact of a peripherally located VFD on the processing of task-relevant facial affective cues.

Pain serves as the focal point of this study for several important reasons. As a ubiquitous and inherently aversive experience that engages both sensory–discriminative and affective–motivational components of the pain matrix (Loeser & Melzack, 1999; Tracey & Mantyh, 2007), faces expressing pain reliably capture attention (Vervoort et al., 2013). Observers decode other’s pain by attending to specific facial cues, such as eye narrowing, brow lowering, nose wrinkling, and upper lip raising (Blais et al., 2019). Beyond its role as a core social signal, pain is particularly relevant in the context of VFDs. Individuals with VFDs, especially when resulting from traumatic injury, frequently experience pain (Sarwer et al., 2022), making pain expressions both ecologically valid and socially meaningful. Thus, pain is not only a physical experience but also a cue that shapes observers’ perceptions, emotional responses, and behavioral intentions (Hadjistavropoulos et al., 2011). Together, these features make pain an especially informative emotional signal for examining how facial differences, such as facial scars, influence social perception and attentional processes.

Thus, the overarching aim of this study is to investigate how the presence of a VFD (i.e., a facial scar) moderates the processing of pain, both in terms of visual processing, as measured by gaze duration, and self-reported pain evaluation. We hypothesized that (i) the visual processing of the face would be influenced by the presence of a peripherally located VFD, resulting in increased gaze toward the VFD and reduced gaze toward internal features; (ii) the evaluation of the pain experience would be biased by the presence of a VFD, such that faces with a VFD would be evaluated as experiencing less pain; and (iii) visual processing and pain evaluation would be correlated, with gaze toward the internal features of the face being positively associated with pain assessment, whereas gaze toward the VFD on peripheral features would be negatively associated with pain assessment.

## 2. Materials and Methods

### 2.1. Participants

Sixty-three students from the University of Caen were recruited. No incentive was provided. Nineteen participants were excluded (six due to having a visible difference, six due to calibration issues, and seven for inferring pain as being due to the scar). Analyses were conducted on the remaining 44 participants (26 females, 18 males) aged between 18 and 29 years (*M* = 19.80, *SD* = 2.17).

An a priori power analysis was conducted via G*Power (Version 3.1.9.6; Faul et al., 2009). The desired sample size was estimated *N* = 36 for a small effect (*f* = 0.25), alpha significance (*α* = 0.05) and a power = 0.95 for repeated-measures ANOVA.

### 2.2. Material

#### 2.2.1. Apparatus

A Tobii Pro Fusion X120 Eye-tracker (SR Research Ltd., Kanata, ON, Canada) was used (sampling rate 120 Hz). The procedure was designed on Tobii Pro Lab (Version 24.21.435; Tobii AB, 2024). The experiment was administered on a laptop.

#### 2.2.2. Stimuli

Participants were exposed to the following sequence: a fixation cross (2 s), a photograph (3 s), and a pain rating scale (until an answer was selected by mouse click). The fixation crosses were adjusted to be placed under the eyes and above the nostril of each photograph. Stimuli consisted of 32 experimental photographs of faces: 2 (with VFD or original version) × 8 (4 women and 4 men) × 2 (pain or neutral). Photographs were extracted from the Pain Emotion Faces Database (PEMF; Fernandes-Magalhaes et al., 2022). The selected photographs were homogeneous in terms of age ([25–29], *M* = 27.25, *SD* = 1.75), pre-evaluated perceived pain intensity ([3.52–5.8], *M* = 4.63, *SD* = 0.80), and pain realism ([57.49–78.82], *M* = 67.85, *SD* = 7.53).

Since our study specifically aimed to investigate the impact of peripherally located VFDs while controlling for potential effects of individual faces, scar-like VFDs were digitally added on the peripheral areas of the face by a graphic designer and photographs were cropped to be 467 × 470 pixels centered within a 2000 × 1125-pixel black frame. Each participant completed 2 training trials (woman without FD; pain, then neutral) before proceeding to the 16 experimental faces. To control for an impact of face or gender, four sets of experimental faces were created. Each set was made of a combination of 16 faces, half with a VFD, half without a VFD; half female, half male. Each face was presented to participants twice (with a neutral and painful expression).^1^

#### 2.2.3. Gaze Duration

**Figure 1:**
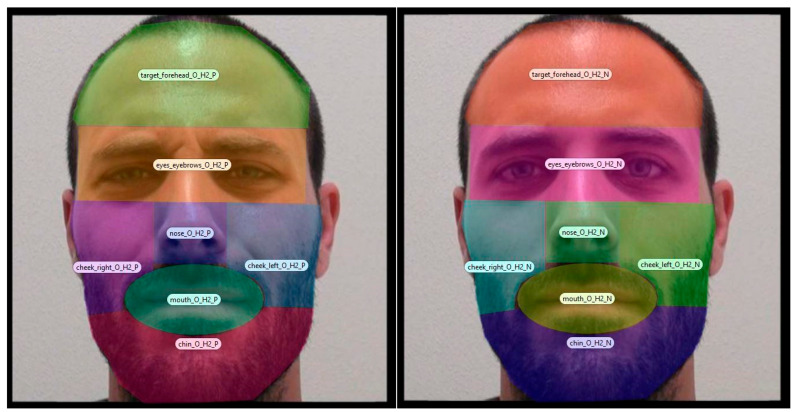
Example of AoI on one of the stimuli. *Note*. The surface and the form of the AoI varied depending on the face, but they were kept constant whether the person on the picture was displaying a VFD or not, and whether they were displaying pain or a neutral expression.

Dwell times were recorded from both eyes of each participant. Dwell time corresponds to the total time (in milliseconds) spent fixating on a specific area of interest (AoI) over a trial. It is an indicator of both processing depth and effort (Rahal & Fiedler, 2019), as well as a common measure in the field (Asaad et al., 2020). This study considered dwell times of two AoIs: one composed of the sum of dwell times on internal features of the face (i.e., eyes and eyebrows, nose, and mouth) and one composed of the sum of dwell times on the peripheral features of the face (i.e., forehead, cheeks, and chin). Each AoI was drawn manually in order to fit photographs the best and not overlap (for an example, see Figure 1).

#### 2.2.4. Pain Rating

Participants were asked to rate the pain level they associated with each photograph using a single-item scale (i.e., “About this person, how much would you rate the pain intensity?”). Answers ranged from 0 (no pain) to 10 (maximal imaginable pain) and were selected by a mouse click.

### 2.3. Procedure

Data collection was carried out in November 2024 at the University of Caen, Normandy. Participants were recruited in person. The study was introduced to them as focusing on face perception and pain severity. Participants completed a consent form and were then randomly assigned to one of the four sets. Past the calibration phase, participants were left alone in the experimental cubicle. Trials were presented in a random order. The demographic questionnaire was administered after the task, followed by a debrief, which included questions about the stimuli.

### 2.4. Statistical Analyses

First, the potential impact of VFDs and pain expression on gaze duration was tested. For that purpose, 2 (face type: original, with visible difference) × 2 (expression: neutral, painful) repeated-measures ANOVAs were run on both eye-tracking variables (i.e., dwell time on the internal features of the face and on the peripheral features of the face). Second, we tested the hypothesis that VFDs and pain expressions would influence pain ratings. To this end, the same 2 × 2 repeated-measures ANOVA was applied to the self-report data. Finally, the relationships between gaze duration and pain rating were investigated through correlations. Thus, eye-tracking and self-report difference scores were calculated by subtracting the mean score for no-pain faces from the mean score for pain faces. All statistical analyses were run on JASP (Version 0.18.1) and Jamovi (Version 2.6) software (JASP Team, 2025; The Jamovi Project, 2025).

## 3. Results

### 3.1. Impact of Visible Differences on Gaze Duration

#### 3.1.1. Peripheral Features

**Figure 2:**
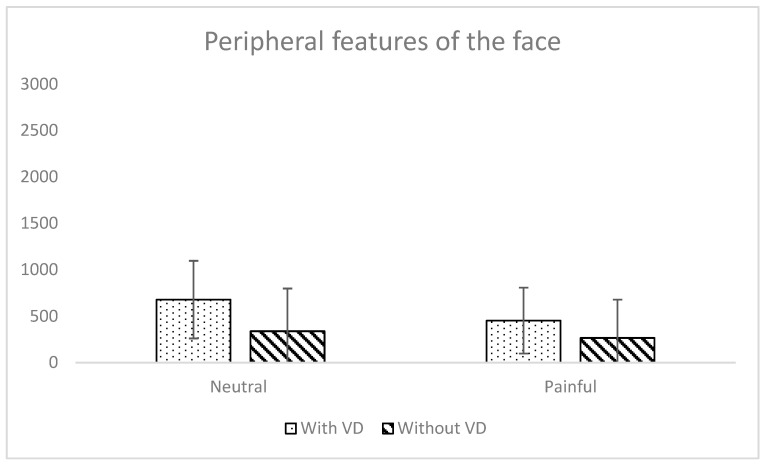
Mean total dwell time occurring in each area, with standard deviations, depending on face type and expression type. *Note*. Both main effects and interaction effect are significant.

A significant main effect of face type was observed on gaze duration directed towards the peripheral features of the face (*F*(1, 43) = 48.50, *p* < 0.001, η^2^_G_ = 0.09; see Table 1 and Figure 2). As expected, participants looked longer at peripheral features when the face had a VFD than when it did not have a VFD. There was also a significant main effect of expression type (*F*(1, 43) = 27.74, *p* < 0.001, η^2^_G_ = 0.03), showing that participants looked longer at peripheral features for neutral conditions than for pain ones. There was also a significant interaction effect between the two independent variables (*F*(1, 43) = 8.73, *p* = 0.01, η^2^_G_ = 0.01). Post hoc analyses revealed, as expected, that participants gazed at peripheral features of the face for a significantly longer duration when the face had a VFD than when it did not have a VFD, the effect being larger for faces displaying a neutral expression (*t*(43) = 7.41, *p*_Bonf_ < 0.001, *d*_Cohen_ = 0.82) than a pain expression (*t*(43) = 4.06, *p*_Bonf_ < 0.001, *d*_Cohen_ = 0.45).

#### 3.1.2. Internal Features

**Figure 3:**
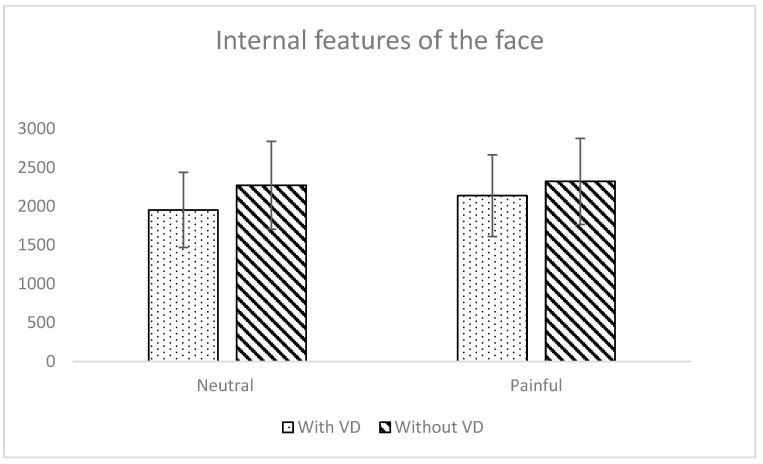
Mean total dwell time occurring in each area, with standard deviations, depending on face type and expression type. *Note*. Only main effects are significant.

We observed a significant main effect of face type on gaze duration towards internal features of the face (*F*(1, 43) = 57.94, *p* < 0.001, η^2^_G_ = 0.05; see Table 1 and Figure 3). As expected, participants looked longer at internal features when the face did not have a VFD than when it did have a VFD. There was also a significant main effect of expression type (*F*(1, 43) = 9.16, *p* < 0.01, η^2^_G_ = 0.01), where participants gazed longer at internal facial features for pain conditions than for neutral ones. No significant interaction between face type and expression was found (*F*(1, 43) = 3.02, *p* = 0.09).

### 3.2. Impact of Visible Difference on Pain Rating

**Figure 4:**
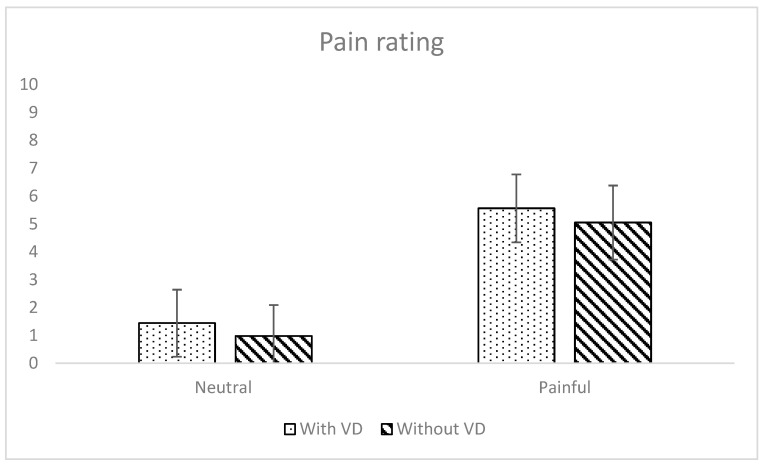
Mean pain ratings, with standard deviations, depending on face type and expression type. *Note*. Only main effects are significant.

A significant main effect of expression on pain rating was observed (*F*(1, 43) = 9.78, *p* < 0.01, η^2^_G_ = 0.04; see Table 1 and Figure 4). As expected, participants rated pain intensity higher for faces displaying a pain expression than a neutral expression. There was a significant main effect of face type on pain rating, but in the opposite pattern than what was expected (*F*(1, 43) = 385.47, *p* < 0.001, η^2^_G_ = 0.74). Participants rated pain intensity higher for faces with a VFD than without a VFD. There was no significant interaction effect between the two independent variables (*F*(1, 43) = 0.06, *p* = 0.81).

### 3.3. Correlations Between Gaze Duration and Pain Rating

None of the expected correlations between visual processing and pain evaluation were found. There was neither a significant positive correlation between gaze toward the internal features of the face and pain assessment, nor a significant negative correlation between gaze toward VFDs on peripheral features and pain assessment.

## 4. Discussion

The present study investigated the influence of a visible facial difference (VFD) on both subjective and oculomotor processing of pain expressions. As expected, gaze patterns were biased toward the area of the visible difference: participants spent less time on internal facial features but looked for a longer duration at peripheral areas when the face had a VFD than when it did not have a VFD. Moreover, the results revealed a moderating effect of facial expression, as the visible difference (in the peripheral area) was gazed at for a shorter duration when the face displayed pain. Contrary to our hypothesis, participants did not rate pain intensity as lower when presented with faces with a VFD. We observed the opposite: the pain ratings were higher for faces with a VFD compared to faces without a VFD. Finally, no significant association was observed between gaze duration and pain rating.

This study replicates previous findings showing that observers are biased by the presence of a visible difference across multiple levels of processing. On the one hand, the modified patterns of gaze exploration observed may correspond to what individuals with VFD often describe as “staring”, an experience known to evoke discomfort and social distress (Bogart et al., 2022). Beyond revealing a heightened visual focus on a distinctive yet task-irrelevant peripheral feature, our findings also indicate that observers visually neglect internal facial regions that convey task-relevant information, i.e., those related to the expression of pain (Blais et al., 2019). By spending less time fixating on these diagnostic features, observers allocated less visual attention to pain-informative facial features in faces with VFD. More generally, while focusing on the VFD, they may lose crucial insight about what the person is actually experiencing (i.e., their thoughts and emotions). This might leave people with VFDs with the impression that they are not seen beyond their differences (Bogart et al., 2012).

Interaction effects indicated that attention to VFDs was reduced when faces expressed pain, suggesting that pain-related information modulates visual processing of faces with VFDs. Moreover, this reduced attention to pain-informative features did not translate into lower estimations of pain. Participants even overrated the pain experience of people with VFDs (compared to people without VFDs), regardless of whether they were displaying pain or a neutral expression. This pattern may stem from VFDs being processed as a perceptual category, i.e., a distinctive category elicited by perceptual cues (Stone, 2021), that activates stereotypes of suffering, like disability (see Nario-Redmond et al., 2019). Indeed, previous work finds that observers show a bias in rating the emotions of people with facial paralysis VFDs as less positive, regardless of the emotion intensity the person with a VFD was actually feeling (Bogart et al., 2014). As such, VFDs might prompt feature-based stereotyping (Hugenberg & Wilson, 2013), with observers expecting people with VFDs to experience more pain, whether they are actually expressing it or not. This could be especially true for VFD associated with a painful event, such as those following traumatic injuries (Sarwer et al., 2022). In line with models of person perception (Fiske & Neuberg, 1990), it is possible that judgments of pain in people with VFDs may have been influenced by stereotypical categorical information rather than solely by individuating information available from the face.

We did not find evidence for any relationship between gaze processing of facial features and pain rating, whether the face was expressing pain or not. Previous research also failed to demonstrate relationships between gaze duration and pain ratings for stimuli without VFDs (Stopyn et al., 2021). In our study, we measured observers’ ratings of pain intensity independently of the targets’ actual pain experience, which would be a more accurate indicator and could, for example, be assessed via self-reported pain. Nonetheless, it is possible that observers do not rely on any specific area of the face when evaluating pain intensity, but focusing on specific areas might be related to more accurate pain perception.

As such, focusing on VFDs might bias empathy for pain, i.e., the ability to share and understand the pain experience of someone else (Goubert et al., 2005). Importantly, however, empathy was not directly assessed in the present study, as participants were not instructed to engage in perspective-taking or affect sharing. Any role of empathy should therefore be interpreted as an indirect, downstream mechanism rather than a primary explanatory factor. One possibility is that reduced attention to pain-diagnostic facial features in faces with VFDs limits access to individuating emotional information, which may constrain cognitive empathy (perspective-taking). Prior eye-tracking work suggests that attention to internal facial features, particularly the eyes, supports accurate emotion decoding and perspective-taking (Cowan et al., 2014). However, the present results indicate that atypical gaze allocation did not translate into lower pain ratings. Instead, participants systematically rated pain higher in faces with VFD, suggesting that judgments relied less on perceptual input and more on top-down expectations.

In the context of disability, visible differences are often associated with stereotypes of vulnerability and suffering, which can shape empathic responses independently of actual emotional expressions (Nario-Redmond et al., 2019). Recent work shows that such stereotypes may lead to biased or paternalistic forms of empathy, characterized by lower affective sharing (Granjon et al., 2023). Notably, Granjon et al. (2023) demonstrated that early affective empathy, as measured via an electro-encephalography (EEG) paradigm, and which can also be interpreted as reflecting attentional biases, was altered when participants viewed faces with disabilities, whereas cognitive empathy remained unaffected. This raises the intriguing possibility that the attentional biases captured via eye-tracking in our study could reflect a similar early, automatic component of empathy, linking perceptual–attentional processes with affective responses to VFD. Future research combining eye-tracking with EEG markers of early and late empathic components would help to disentangle these processes and clarify how attention and empathic processing interact in the social perception of VFDs.

While highlighting biases in pain assessment of people with VFDs, this study has practical implications. Experiences of VFDs and pain can be interconnected, either because VFDs emerge as a result of a painful event (e.g., traumatic injury, burn; Sarwer et al., 2022) or because people undergo painful surgery to reduce functional difficulties and visible difference (e.g., Feragen et al., 2022). It is crucial for people with visible differences that their pain experience is accurately evaluated. This is true in healthcare settings, where pain evaluation can be especially challenging (Kappesser & De Williams, 2002), but also in educational or workplace settings, where pain legitimacy can impact available accommodations (e.g., Wakefield et al., 2018). Importantly, one risk for people with VFDs is that their pain may be misestimated due to a focus on their difference.

This study has limitations. The stimuli were scars, which can be perceived as resulting from a painful injury. As this research was specifically addressing biases in pain perception, we removed data from participants that disclosed having inferred pain as resulting from the scar during the debriefing session (the question was systematically asked by the investigator). As such, this resulted in data loss from six participants. Future studies should vary the type of VFD features in order to generalize the findings across different facial differences, including differences that may imply less pain, such as birthmarks. In addition, they should also consider varying the type of emotion. Indeed, we could expect VFDs to be a perceptual category that primes pain (or negative emotions; Bogart et al., 2014) to a larger extent than joy (or other positive emotions; Jamrozik et al., 2019). Finally, we focused on peripherally located VFDs; however, future research would benefit from investigating the impact of VFDs located on internal facial features (e.g., cleft lip) as well. If VFDs systematically bias the visual processing of individuating information regardless of their location, eye-tracking alone may fail to distinguish visual processing directed toward the VFD and that directed toward the internal facial features. Complementary methodological approaches would therefore be needed and could also help address other limitations of the present research.

## 5. Conclusions

This research shows that the presence of VFDs influences observers’ visual facial processing and their pain ratings. The findings are consistent with the idea that the processing of faces with VFDs may shape how observers interpret facial information. By documenting these patterns, the present study contributes to identifying factors that may influence evaluations of faces with VFDs. These results point to directions for future research aimed at better understanding and, ultimately, reducing misperceptions in social interactions involving individuals with facial differences.

## Figures and Tables

**Table 1:** Means (standard deviations) of dwell time (in ms) for each AoI and of pain rating depending on face type and expression type.

		Expression Type		
		Neutral	Painful	All
	**Face type**			
**Dwell time:** **Peripheral features of the face**	**With VFD**	678.32 (418.06)	452.37 (355.09)	565.35 (381.03)
	**Without VFD**	337.33 (461.40)	265.70 (412.42)	301.52 (436.91)
	**All**	507.83 (439.73)	359.03 (383.76)	
	**Face type**			
**Dwell time:** **Internal features of the face**	**With VFD**	1953.03 (485.04)	2137.45 (527.48)	2045.24 (506.26)
	**Without VFD**	2270.97 (567.99)	2321.97 (554.83)	2296.47 (561.41)
	**All**	2112.00 (526.52)	2229.71 (541.16)	
	**Face type**			
**Pain rating**	**With VFD**	1.44 (1.20)	5.56 (1.22)	3.50 (1.21)
	**Without VFD**	0.98 (1.11)	5.05 (1.33)	3.02 (1.22)
	**All**	1.21 (1.16)	5.31 (1.28)	

## Data Availability

All data and materials have been made publicly available via the Open Science Framework and can be accessed at this link: https://osf.io/bh4ek/. The design and analysis plans for the experiments were preregistered at the Open Science Framework and can be accessed at https://osf.io/bh4ek/overview?view_only=81c67f845fc748d2b7962e42cca19d27.

## Note

1 The stimuli for each variation have been made publicly available online at: https://osf.io/bh4ek/files.
